# Supplementary material for: BALAD-2 Emerges as the Most Accurate Prognostic Model in Hepatocellular Carcinoma: Results from a Biobank-Based Cohort Study
Source: Cancers (Basel). 2025 Oct 28;17(21):3457. doi: 10.3390/cancers17213457 (PMC12610203; doi:10.3390/cancers17213457)
Supplement: Supplementary file 1 [file cancers-17-03457-s001.zip › cancers-3919948-supplementary.pdf]

Supplementary Table S1. Univariate Analysis of Variables Associated with Overall Survival

	p-value
**Age**	0.526
**Male gender**	0.079
**Body-mass index**	0.410
**Diabetes mellitus**	0.332
**Hypertension**	0.320
**Hyperlipidemia**	0.165
**Viral etiology (vs. non-viral etiology)**	*0.750*
**Cirrhosis**	*0.159*
**CTP score**	<0.001*
**MELD**	<0.001*
**ALBI**	<0.001*
**Esophageal varices**	0.124
**History of variceal bleeding**	0.011*
**Creatinine**	0.190
**Platelet count**	0.555
**AST**	<0.001*
**ALT**	0.013*
**ALP**	<0.001*
**GGT**	<0.001*
**Sodium**	<0.001*
**Portal vein thrombosis**	<0.001*
**Maximum tumor size**	0.002*
**Number of tumoral lesions**	<0.001*
**Extrahepatic metastasis**	<0.001*
**Treatment category**	<0.001*
**AFP**	<0.001*
**AFP-L3**	<0.001*
**DCP**	<0.001*
**GALAD**	<0.001*
**ASAP**	<0.001*
**GAAP**	<0.001*
**BALAD**	<0.001*
**BALAD-2**	<0.001*
**DOYLESTOWN**	<0.001*
**aMAP**	0.048*

AFP: Alpha-fetoprotein; AFP-L3: Lens culinaris agglutinin-reactive alpha-fetoprotein; ALT: Alanine aminotransferase; ALP: Alkaline phosphatase; AST: Aspartate aminotransferase;
CTP: Child-Turcotte-Pugh, DCP: Des-gamma-carboxy prothrombin; DM: Diabetes mellitus; GGT: Gamma-glutamyl transferase
